# Nucleation and manipulation of single skyrmions using spin-polarized currents in antiferromagnetic skyrmion-based racetrack memories

**DOI:** 10.1038/s41598-022-19587-6

**Published:** 2022-09-08

**Authors:** Hamza Belrhazi, Mohamed El Hafidi

**Affiliations:** Condensed Matter Physics Laboratory, Department of Physics, Faculty of Science Ben M’sik, Hassan II University of Casablanca, D. El Harty Av., B.P 7955, 20165 Casablanca, Morocco

**Keywords:** Spintronics, Magnetic properties and materials

## Abstract

In this work, an ultrafast nucleation of an isolated anti-ferromagnetic (AFM) skyrmion was reported in an AFM layer with DMi strengths of 0.47$-$0.32 $\mathrm{mJ}/{\mathrm{m}}^{2}$ using spin-transfer torque by locally injecting pure spin currents into magnetic tracks. Besides, we revealed the key advantages of AFM skyrmion-based racetrack memories by comparing the motion of AFM and FM skyrmions driven by spin–orbit torques (SOTs) for different skyrmion sizes along racetrack memories with various notch sizes. Our results indicate that for AFM skyrmion, the skyrmion Hall effect does not exist during the skyrmion motion, therefore at small skyrmion sizes, we succeeded to overcome the repulsive forces developed in the notch area for low and large SOTs. The obtained findings were carefully analyzed by computing the variation of energy barriers associated with the notch for different skyrmion sizes using minimum energy path (MEP) calculations. We showed that the larger the skyrmion size, the harder it is to shrink the skyrmion in the notch which produces a high energy barrier (E_b_) for large skyrmion sizes. Moreover, as the notch size increases, the skyrmion size shrinks further, and hence E_b_ increases proportionally. Nevertheless, we proved that AFM skyrmions are more efficient and flexible than FM skyrmions against boundary forces.

## Introduction

Magnetic skyrmions are topologically nontrivial nanoscale spin-textures having particle-like properties in continuous field theory^1^. They can be nucleated in both bulk non-centrosymmetric magnetic materials and ultrathin films^1,2^ due to the symmetry breaking together with strong spin–orbit coupling. In the presence of Dzyaloshinskii–Moriya interaction (DMi), magnetic skyrmions can be stabilized in ferromagnets with a fixed chirality which implies a definite skyrmion number (Q =  ± 1)^3^. Magnetic skyrmions have attracted considerable attention because of their high potential to handle information at low energy consumption and/or high processing speed due to their topological properties and controlled size^4,5^. Additionally, the ability to solve intrinsic pinning issues that appear throughout the Domain Walls (DWs) motion by avoiding local structure imperfections caused by Magnus forces offers a promising benefit for skyrmion-based devices^6^. Low threshold current density requirements at local pinning sites are a key advantage for skyrmion-based racetrack memory and logic gate devices, featuring high processing speed, ultrahigh-density magnetic data storage, and low power consumption^4,7,8^. Thus, magnetic skyrmions are considered as a robust information carrier in the next generation of highly functional spintronics nanotechnology.

Magnetic skyrmions were successfully manipulated with various forces either by applying a spin-polarized current (spin-transfer torque (STT)^9^ or spin–orbit torque (SOT)^10^), short laser pulse, or a pure electric field^11,12^ in multilayer systems. However, driving skyrmions by SOTs along a racetrack may cause them to deviate from the longitudinal motion due to the occurrence of a transverse velocity component during the skyrmion motion so-called skyrmion Hall effect (SkHE)^13^. The strength of SkHE is related to the skyrmion Hall angle ${\theta }_{SkHA}$, which is derived from the ratio between the transverse velocity component and the longitudinal velocity component (${\theta }_{SkHA}$ ~${v}_{t}/{v}_{l}$), therefore, reducing SkHE means reducing ${\theta }_{SkHA}$. Since its topological number is ± 1, the direction of the transverse motion can be predicted by the topological number of skyrmion Q. For small spin-polarized currents, SkHE can be neglected (${\theta }_{SkHA}$~$0$), but under large currents, this effect increases as well^14,15^. In flat racetracks, the skyrmion dynamics under large currents are significantly affected by SkHE, where the strong transverse velocity component drives the skyrmion to the racetrack boundary which leads to annihilate the skyrmion due to the sensitivity of skyrmion to edge roughness^16^. However, if the edge roughness of the racetrack memory is small enough, the skyrmion motion under low spin-polarized currents can be moved closer to the edge along the racetrack^16^. For emerging applications, high-speed skyrmions along the racetrack are always required thus, SkHE poses a crucial problem for the development of fast, small, and low-energy consumption devices for information computing and processing^17^. This challenge motivated many researchers to develop new methods, as there have been many papers reporting important approaches and designs for exploiting or eliminating the SkHE^18,19^. For instance, the racetrack memory based on the synthetic antiferromagnets (SAF) provides a perfect hosting for skyrmion, where the skyrmion number of two coupled skyrmions in the SAF cancel each other therefore the topological number equals zero (${\theta }_{SkHA}$~0)^18^. Other works have suggested changing the geometry entirely by using racetrack memories shaped as nanotubes^19^ instead of typical flat tracks, because there are no boundaries in the tangent direction and therefore skyrmion cannot annihilate.

Recently, systems based on the anti-ferromagnet (AFM) emerge as a much better alternative to FM for hosting skyrmions in non-volatile storage devices^20–22^ due to the following reasons: they are characterized by zero Magnus force which completely suppresses SkHE^21^, therefore the velocity component of AFM skyrmion motion can only develop along the longitudinal direction, which allows the skyrmion to move for long distance without touching the racetrack edges. As a result, AFM skyrmions release from the limitations of the maximum spin-polarized current, making them a very good candidate for racetrack memory and logic gate devices^20,21^. In the ground state, the 2D AFM layer consists of two coupled sub-lattices (A) and (B) that are completely polarized to each other. The 2D topological soliton (AFM skyrmion) can also be nucleated, moved, and converted into an AFM DW pair in AFM systems^21,23^ either by STTs, SOTs, or spin waves^24–26^ as the FM skyrmion. Furthermore, the processing speed of AFM skyrmion is much higher than that of FM skyrmion^23^. Additionally, the AFM skyrmion-based STNO (spin-torque nano oscillator) produces ultrahigh oscillation frequencies (THz regime) much higher as compared to FM skyrmion-based STNO^27^.

Practically, we usually pattern the racetrack with notches as geometrical constructions to facilitate the creation of skyrmions^28–30^. However, the notched racetrack can also inhibit SOT-induced skyrmion motion as well as limit the maximum spin-polarized current due to the width of the geometrical construction^31,32^. To solve this dilemma, several methods have been proposed to handle this issue. Upadhyaya et al. proved that skyrmion trajectory can be controlled by applying an electric field in a certain pattern. Thus, skyrmions can be guided in the desired trajectory along the racetrack memory^33^. More recently, Jiang et al. proposed a novel device designed in the form of four-terminals in a nano-racetrack to separate the skyrmion generation line from the motion line, therefore we generate and accelerate the skyrmion separately along the racetrack memory^31^.

Motivated by this state of affairs, we numerically investigated in this manuscript the nucleation of an isolated AFM skyrmion at low temperature by locally injecting a pure spin current perpendicularly to an AFM monolayer using micromagnetic simulation. Besides, we revealed the key advantages of the AFM skyrmion motion in comparison with the FM skyrmion driven by low and large SOTs along racetrack memories for different skyrmion sizes. Furthermore, we reviewed the impact of geometrical construction on skyrmion motion for various notch sizes. The paper is organized as follows. In Section II, we present the software and tools used for simulation and discuss the properties of the AFM skyrmion, then we put down the micromagnetic model with the theoretical formalism adopted. In Section III, we carefully analyze and discuss the main results. Finally, we highlight the main conclusions and give some perspectives in Section IV.

## Method

**Figure 1:**
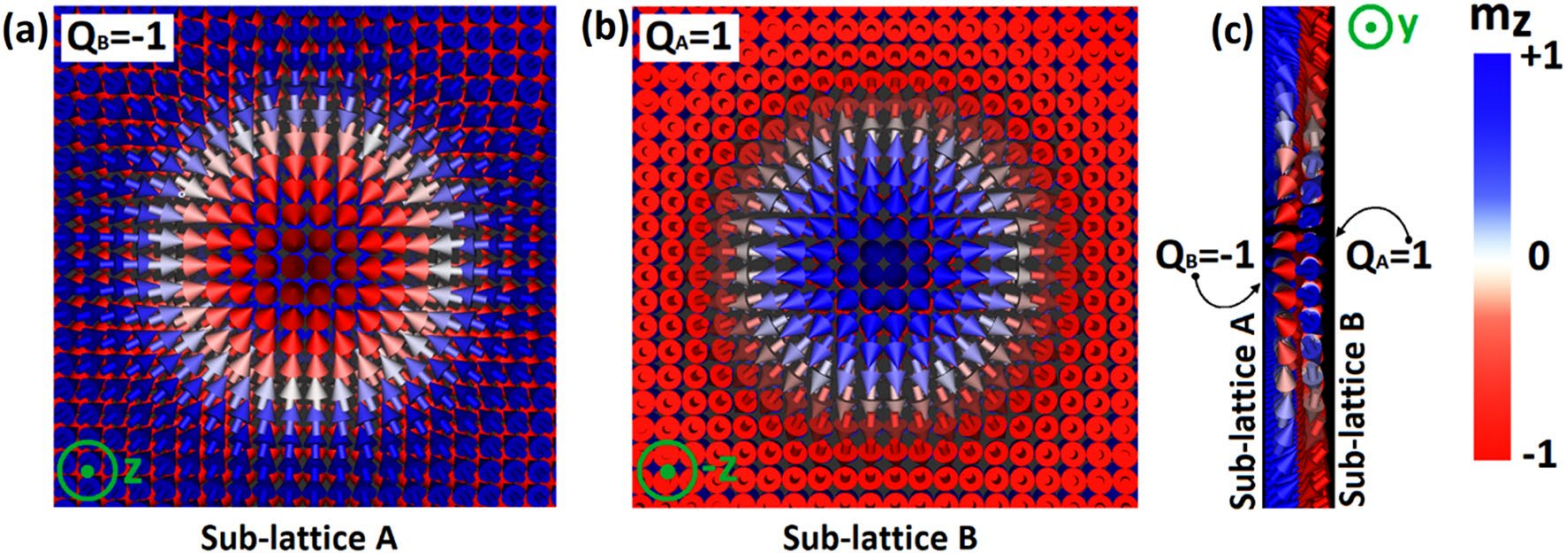
Top view of two sub-lattices (A) and (B) in an AFM monolayer within AFM skyrmion defined with opposite skyrmion numbers (**a**) ${{\varvec{Q}}}_{{\varvec{A}}}$ $=$ $+1$ and (**b**) ${{\varvec{Q}}}_{{\varvec{B}}}\boldsymbol{ }=\boldsymbol{ }-1$. (**c**) Lateral view of the skyrmion in an AFM monolayer sliced along the y-axis into the skyrmion core.

Micromagnetic simulations were performed using the Mumax3 Framework together with the Ubermag package^34^ to reproduce and analyze magnetic nanotextures in an AFM system as shown in Fig. 1. We point out that Mumax3 is a GPU-accelerated micromagnetic program based on the Nvidia CUDA toolkit, allowing it to achieve higher performance and deal with larger scales as well^34^. We employed Mumax3 to investigate the space and time-dependent magnetization characteristics by solving the Landau-Lifshitz-Gilbert equation on a finite-difference grid. For samples with tiny cell sizes, Mumax3 is analogous to the atomistic model, and hence it is adequately appropriated to the AFM system. Accordingly, we successfully modeled the AFM system by using the lattice Hamiltonian (See supplementary movie 1), which is expressed as:

1 $${H}_{AFM}={J}_{\mathrm{ext}}\sum_{<i,j>}{{\varvec{m}}}_{{\varvec{i}}}{.{\varvec{m}}}_{{\varvec{j}}}-{k}_{a}\sum_{i}{{({{\varvec{m}}}_{\boldsymbol{ }}}_{{\varvec{i}}}^{{\varvec{z}}})}^{2}+\sum_{<i,j>}{{\varvec{d}}}_{{\varvec{a}}}.({{\varvec{m}}}_{{\varvec{i}}}{\times {\varvec{m}}}_{{\varvec{j}}}).$$

The lattice Hamiltonian includes the contribution of AFM exchange interaction with ${J}_{\mathrm{ext}}$ $\mathrm{is}$ the AFM exchange constant whilst the last two term describe the contributions of perpendicular magnetic anisotropy (PMA) and DMi in the atomistic model wherein ${k}_{a}$ and ${{\varvec{d}}}_{{\varvec{a}}}$ are the anisotropy constant and DMi vector, respectively.

The skyrmion number is written as^35^:

2 $${Q}=-\frac{1}{4\pi }\int {d}^{2}x {\varvec{m}}\left({\varvec{x}}\right).\left({\partial }_{x}{\varvec{m}}\left({\varvec{x}}\right)\times {\partial }_{y}{\varvec{m}}\left({\varvec{x}}\right)\right).$$

The total topological number of an AFM skyrmion is zero. However, the topological number of AFM skyrmion ${\mathrm{Q}}_{\mathrm{AFM}}$ associated with sub-lattices A and B can be given by the discretized version that determines the opposite core polarities of skyrmion (${Q}_{A}$ $=-$ ${Q}_{B}=+$ 1)^23^:

3 $${{Q}_{AFM}}_{\tau }=-\frac{1}{4\pi }\sum_{ijk}{{\varvec{m}}}_{{\varvec{i}}}^{{\varvec{\tau}}}.\left({{\varvec{m}}}_{{\varvec{j}}}^{{\varvec{\tau}}}\times {{\varvec{m}}}_{{\varvec{k}}}^{{\varvec{\tau}}}\right),\uptau =\mathrm{A},\mathrm{B}$$

Thanks to its topological protection and the AFM exchange interaction, AFM skyrmion cannot be separated into individual skyrmions, again ${Q}_{AFM}$ cannot change even when the AFM skyrmion is deformed.

The time-dependent magnetization behavior is given by solving the generalized Landau-Lifshitz-Gilbert (LLG) equation (Eq. 4) with additional terms of Spin–Orbit Torque (SOT) originating from the spin Hall effect (SHE) in the heavy metal (HM) and Spin-Transfer Torque (STT) stems from the pure spin current out-of-plane^36,37^:

4 $$\frac{{d{\varvec{m}}}_{{\varvec{i}}}}{dt}=-{\upgamma }_{0}\left({{\varvec{m}}}_{{\varvec{i}}}\times {{{\varvec{H}}}_{{\varvec{i}}}^{{\varvec{e}}{\varvec{f}}{\varvec{f}}}}\right)+\mathrm{\alpha }\left({{\varvec{m}}}_{{\varvec{i}}}\times \frac{{d{\varvec{m}}}_{{\varvec{i}}}}{dt}\right)+{\tau }_{SOT}+{\tau }_{STT}.$$

where ${m}_{i}$ represents magnetic moments of each sub-lattice (where, $i$ = A, B) and ${H}_{i}^{eff}$ is the effective field. γ and α are respectively the gyromagnetic ratio of the electron and the damping constant. The first and second terms in Eq. (4) are the precession and damping term, respectively. The additional terms represent the damping-like SOT^38,39^ and STT out-of-plane^34,40,41^:

5 $${\tau }_{SOT}=-{\upgamma }_{0}{{\varvec{m}}}_{{\varvec{i}}}\times \left({{\varvec{m}}}_{{\varvec{i}}}\times {\frac{\hslash {\theta }_{SHE}{j}_{HM}}{2{\mu }_{0}e{M}_{s}t}}{{\varvec{e}}}_{{\varvec{y}}}\right), {\tau }_{STT}={-\upgamma }_{0}\beta \times \left({{\varvec{m}}}_{{\varvec{i}}}\times {{\varvec{e}}}_{{\varvec{z}}}\times {{\varvec{m}}}_{{\varvec{i}}}\right).$$

where ${\theta }_{SHE}$ is the spin Hall angle and ${j}_{HM}$ represents the current density flowing in the HM layer with $t$ and *e* are the thickness of the AFM monolayer and the electron charge, respectively. While $\beta$ represents the STT strength ($=\frac{J\hslash p}{2et{M}_{s}}$) and ${\varvec{p}}$ is the polarization direction of electrons $({\varvec{p}}=-{{\varvec{e}}}_{{\varvec{z}}})$.

**Figure 2:**
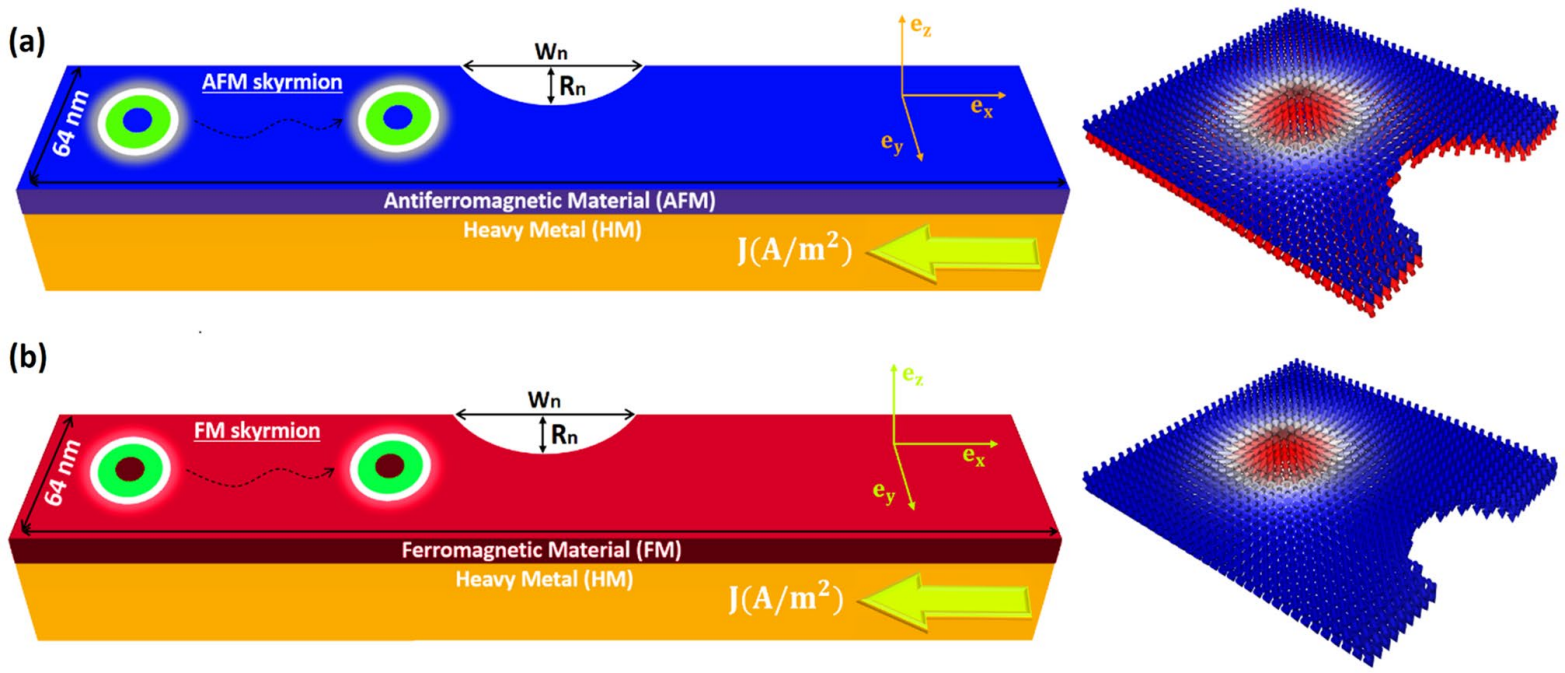
Illustration of two bilayer systems consisting of AFM/HM and FM/HM designed as a racetrack memory notched at the center and traversed by an electric current along the x-axis.

We point out that the damping-like SOT in Eq. (5) is not explicitly implemented in Mumax3, Thus, we implemented this torque in Mumax3 by using the custom fields functionality to add it as an effective field term as shown in Eq. (5). We first defined the constants and then added the damping-like SOT term using the function AddFieldTerm () to generate the damping-like SOT. Besides, we assumed a material with positive spin Hall angle (${\theta }_{SHE}$>0) and a current pulse in $-$ x direction along the HM layer. We considered an AFM skyrmion hosted in an AFM thin film-shaped as a racetrack memory of dimensions 270 nm × 64 nm × 2 nm. The periodic boundary conditions are also adapted along the $x$*-*axis to avoid annihilation of AFM at the end of the nanoracetrack (See supplementary movie 5) then we stabilized the AFM skyrmion on the left of the racetrack with DMi together with PMA stem from the SOC provided by a HM (W, Ta, Pt…). In Fig. 2, we presented our racetracks consisting of AFM/HM and FM/HM bilayers with a circle segment (notch) at the center defined by the notch size of W_n_ and R_n_.

The characteristic parameters of the material used in the micromagnetic simulation are given as follows: the spin Hall angle ${\uptheta }_{\mathrm{SHE}}=$ $0.15$, the Gilbert damping coefficient $\alpha =0.25$, the gyromagnetic ratio $\gamma =-2.21\times 1{0}^{6}\mathrm{ m}/\mathrm{As}$, the saturation magnetization for each sub-lattice ${\mathrm{M}}_{\mathrm{s}}=0.29\times 1{0}^{6}\mathrm{A}/\mathrm{m}$, the exchange stiffness ${\mathrm{A}}_{\mathrm{ext}}=-1.3\times 1{0}^{-11}\frac{\mathrm{J}}{\mathrm{m}}\left(=\frac{2{J}_{\mathrm{ext}}{\mathrm{S}}^{2}}{\mathrm{a}}\right)$^42,43^ and the PMA constant $\mathrm{K }=0.8\times 1{0}^{6}\mathrm{ J}/{\mathrm{m}}^{3}(={\mathrm{k}}_{a}{\mathrm{n}}_{\mathrm{at}}/{\mathrm{a}}^{3})$^44^ and $\mathrm{D }=0.47-0.32\mathrm{ mJ}/{\mathrm{m}}^{2} (=3\sqrt{2}{\mathrm{d}}_{a}/({\mathrm{N}}_{F}{\mathrm{a}}^{2}))$^45^. Here, S is the spin quantum number and a is the unit cell size (m) while ${\mathrm{n}}_{\mathrm{at}}$ and ${\mathrm{N}}_{F}$ are the number of atoms per unit cell and the number of magnetic layers, respectively. It is worthwhile to mention that the magnetic parameters used in this study are in the same order as those of AFM materials. We refer that the samples are divided into cubical cells of size 1 nm × 1 nm × 2 nm which is smaller than the exchange length ${l}_{ex}$ = 4.03 nm $(=\sqrt{|{\mathrm{A}}_{\mathrm{ext}}|/\mathrm{K}})$ to ensure reasonable numerical accuracy in the relaxation process and time-dependent magnetization dynamics.

## Results and discussion

**Figure 3:**
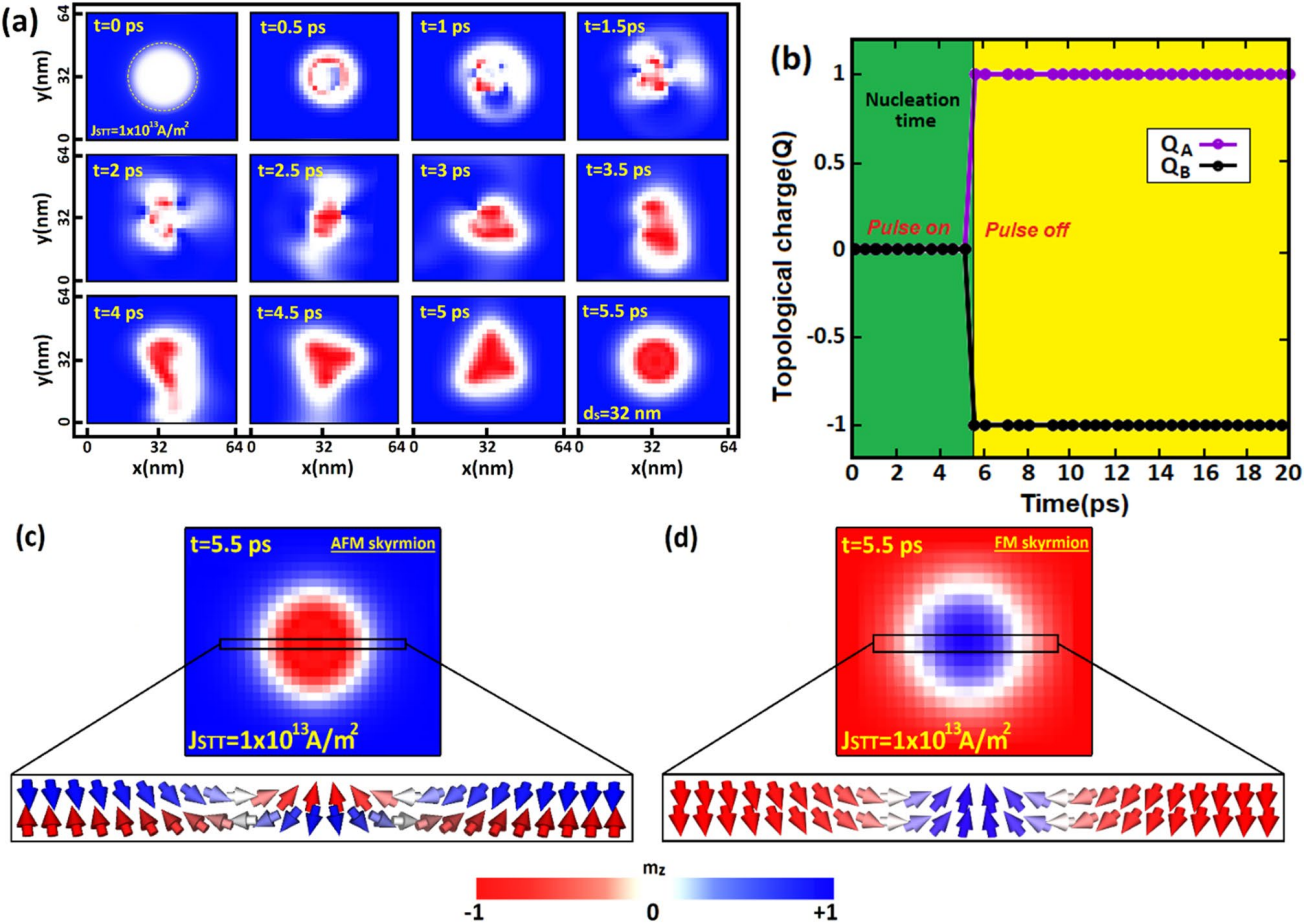
(**a**) Snapshots of the nucleation steps of isolated AFM skyrmion using a vertically injected spin-polarized current (STT) perpendicular-to-plane (CPP) of the 2D AFM layer in a circular region (yellow circular). (**b**) The time evolution of topological charge Q_A_ and Q_B_ during the nucleation. (**c,d**) Snapshots of isolated AFM and FM skyrmions after the nucleation time.

As stated before, we aim in this section to present and discuss the main numerical results obtained by using the micromagnetic simulation. Our purpose consists in investigating first the nucleation of an isolated AFM skyrmion by locally injecting a pure spin (spin-polarized) current perpendicular-to-plane of a 2D AFM layer. In Fig. 3a, we nucleated an AFM skyrmion in a 2D layer with an AFM ground state via spin-transfer torque (STT) by locally injecting a pure current perpendicular-to-plane (CPP) of the racetrack memory (See supplementary movie 2). We began with an AFM configuration with DMi strength of 0.45 mJ/m^2^ then we injected a perpendicular spin-polarized current of $1\times {10}^{13}\mathrm{A}/{\mathrm{m}}^{2}$ towards a circular region in the AFM layer defined with a diameter of d $=$ 45 nm as indicated at 0 ps in Fig. 3a. We continue to inject the spin-polarized current perpendicularly into the current-injected region. Subsequently, we observe at 0.5 ps that the STT induces spin-flip in the target area, resulting in spin precessional motion in the AFM material. At 5.5 ps, we switched off the current pulse and stabilized the energies by relaxing the system to minimize the DMi and AFM exchange energies. After the relaxation time, we succeeded to stabilize numerically the AFM thin film with an AFM skyrmion observed in the target area with an ultrafast nucleation time reaching 5.5 ps and a skyrmion diameter of ${\mathrm{d}}_{\mathrm{s}}=35$ nm (see Fig. 3a). In Fig. 3b, we plotted the time development of the topological charges ${\mathrm{Q}}_{\mathrm{A}}$ and ${\mathrm{Q}}_{\mathrm{B}}$ corresponding to the sub-lattice (A) and sub-lattice (B) in the AFM monolayer during and before the nucleation time. As we notice in the nucleation process, the topological charges equal zero for both sublattices. However, once the current pulse is turned off, we observe the creation of the AFM skyrmion, therefore the topological charge develops suddenly to a fixed value (${\mathrm{Q}}_{\mathrm{A},\mathrm{B}}=\pm 1$) after an ultrafast nucleation time of 5.5 ps which confirms the topological characteristic of the AFM skyrmion. It should be noticed that the relaxation period was not included in Fig. 3. We illustrated in Fig. 3c the final configuration of the AFM skyrmion obtained numerically by a perpendicular spin-polarized pulse of $1\times {10}^{13}\mathrm{A}/{\mathrm{m}}^{2}$ with a lateral view of the AFM skyrmion spin texture sliced along the y-axis into the skyrmion core (See supplementary movie 3). Similarly, we nucleated the FM skyrmion in a FM monolayer by applying a perpendicular spin-polarized current of $1\times {10}^{13}\mathrm{A}/{\mathrm{m}}^{2}$ towards a circular area in the FM layer defined with a diameter of d $=$ 45 nm (Fig. 3d). It is noteworthy to mention that different nucleation times were tested. As a result, we found that for long nucleation times, the AFM skyrmion did not reveal in these cases; instead, the spin-polarized current induced large chiral DWs along the y-axis. For short nucleation times (below 5.5 ps), the system does not acquire enough energy to generate AFM skyrmions, and hence, the spin direction of each sublattice returns to its initial orientation after minimizing the DMi and AFM exchange energy.

**Figure 4:**
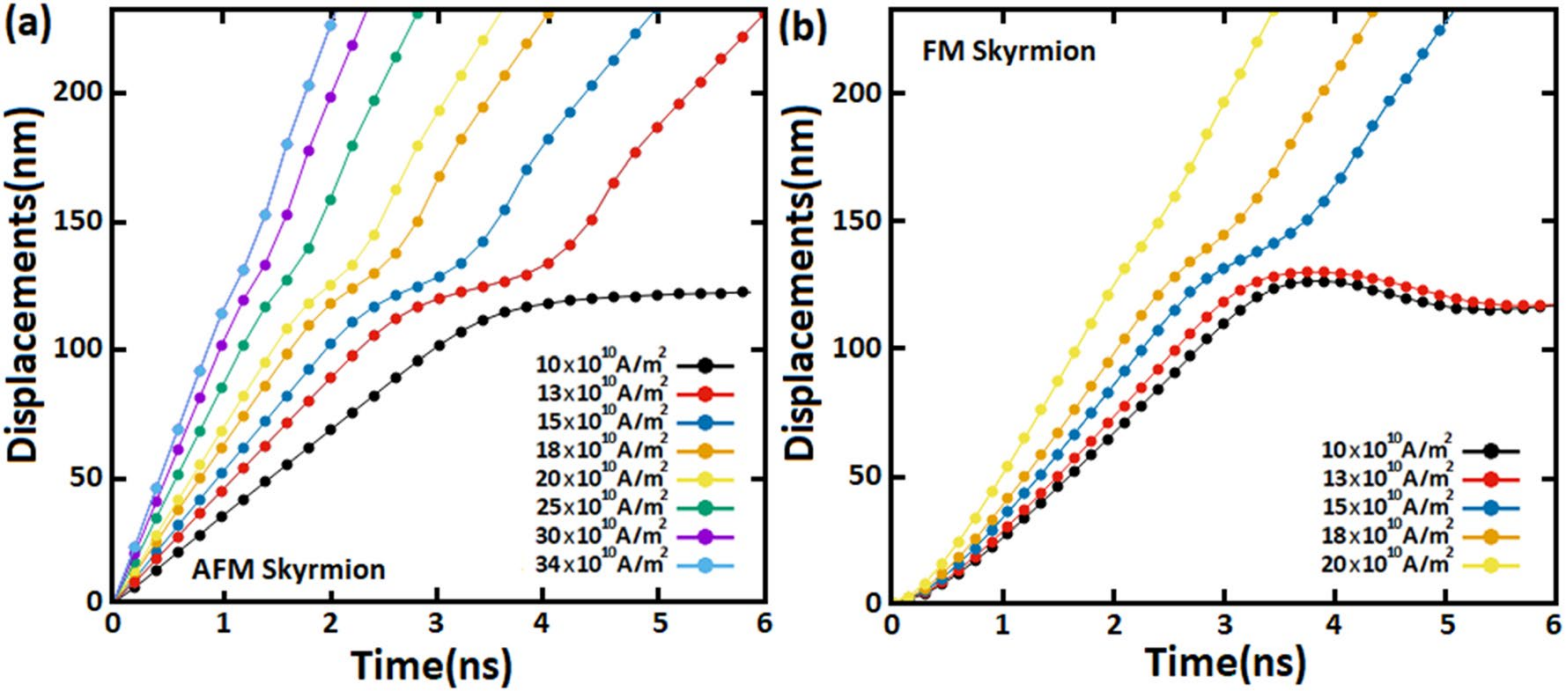
Displacements of (**a**) AFM and (**b**) FM skyrmions by various spin-polarized currents (SOTs) along racetrack memories with a notch size of ${\mathbf{R}}_{{\varvec{n}}}=$ 18 nm and ${\mathbf{W}}_{{\varvec{n}}}=$ 50 nm.

**Figure 5:**
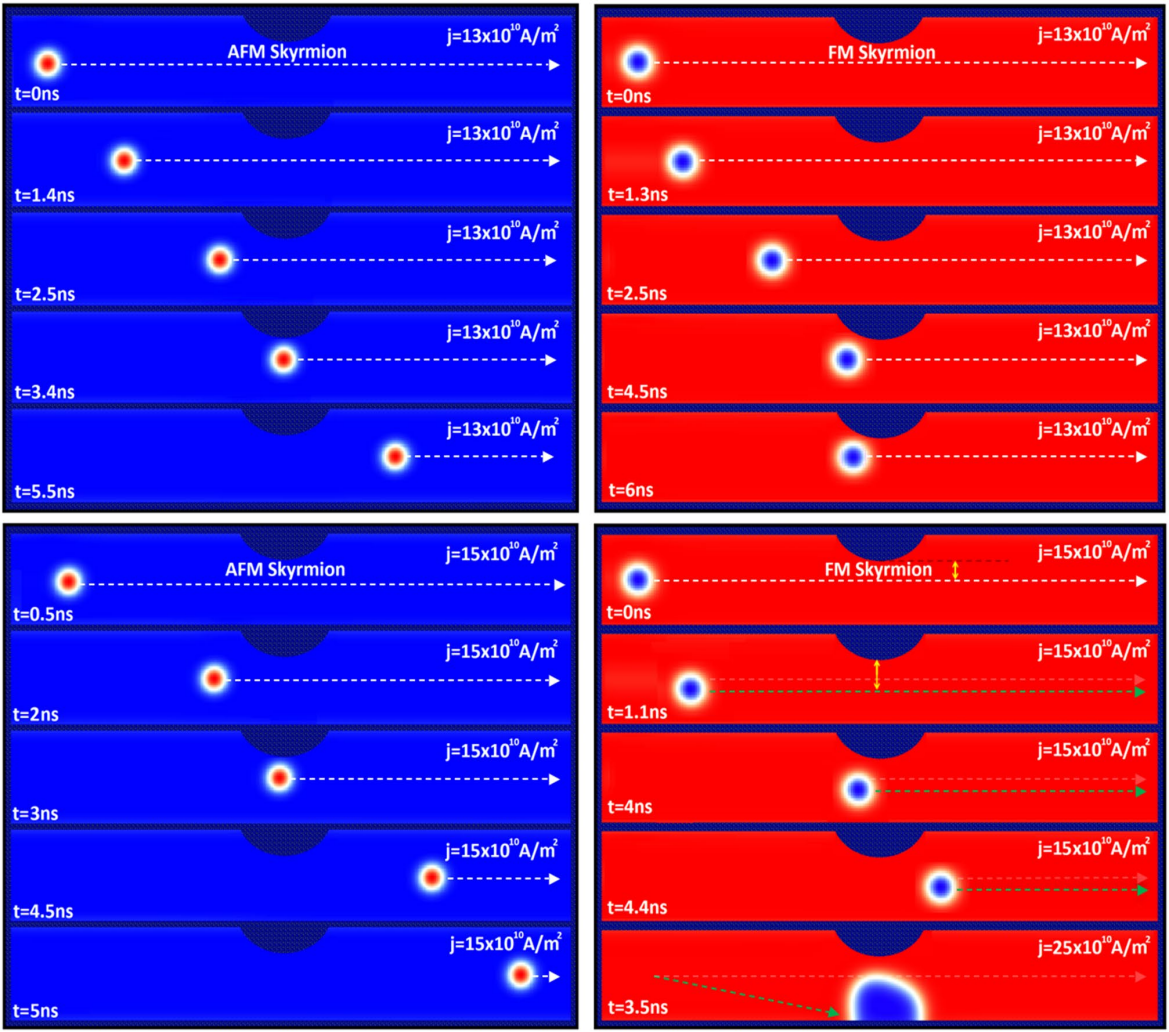
Snapshots of the AFM and FM racetracks during the displacements of AFM and FM skyrmions under different spin-polarized currents (SOTs) with a notch size of ${\mathbf{R}}_{{\varvec{n}}}$=18 nm and ${\mathbf{W}}_{{\varvec{n}}}$=50 nm.

As shown in Fig. 4, we investigated the displacement of AFM skyrmion (Fig. 4a) and FM skyrmion (Fig. 4b) by SOTs along a racetrack memory notched with a notch size of ${\mathrm{R}}_{n}=$ 18 nm and ${\mathrm{W}}_{n}=$ 50 nm. Far from the notch, skyrmions move steadily towards the $+x$ direction with different velocities according to the SOT value. However, we observe that the AFM and FM skyrmions get stuck next to the notch under a low spin-polarized current of 10 $\times {10}^{10}\mathrm{A}/{\mathrm{m}}^{2}$ due to the repulsive forces. Nevertheless, in the presence of sufficiently large spin-polarized currents, SOTs may drive the AFM skyrmion to exceed this geometrical construction. For instance, the AFM skyrmion can exceed the notch for the SOT induced by applying a current density of 13 $\times {10}^{10}\mathrm{A}/{\mathrm{m}}^{2}$. The ability to exceed the notch is predicted through the competition between the boundary forces generated by the geometrical structure of the notch and the SOT used along the racetrack memory. We illustrated this competition by the deflections in the displacement plots, as shown in Fig. 4, resulting in the skyrmion motion decelerating in the notch area before returning toward the normal path after passing through the notch. For large currents, we observe that the higher current, the smaller deflection therefore the velocity remains almost constant throughout the skyrmion motion along the track memory. Whereas for FM skyrmion, even increasing the spin polarization current to 13 $\times {10}^{10}\mathrm{A}/{\mathrm{m}}^{2}$, it is clear that the SOT cannot overcome the repulsive forces, and hence the skyrmion cannot exceed the notch. This result is basically due to the skyrmion size in the FM track which is somewhat larger than the AFM skyrmion, and thus the repulsive forces are rather decisive in this case. However, this issue can be avoided by increasing the spin-polarized currents to specific values of 15 $\times {10}^{10}\mathrm{A}/{\mathrm{m}}^{2}$, 18 $\times {10}^{10}\mathrm{A}/{\mathrm{m}}^{2}$, and 20 $\times {10}^{10}\mathrm{A}/{\mathrm{m}}^{2}$ where the transverse velocity component leads the FM skyrmion away from the notch, which reduces the repulsive forces (Fig. 5). As a result, the FM skyrmion keeps moving next to the edge under these current densities without being annihilated. Although, it should be noted that the SkHE grows with the spin-polarized currents in the FM racetrack. Therefore, for large current densities, SkHE drives FM skyrmions to annihilate at the track edge. We point out that if we consider the notch at the opposite edge, the FM skyrmion will either annihilate or stop at the notch depending on SOT strengths, because the Magnus force drives the skyrmion to the notch which contributes to an increase in repulsive forces between the notch and skyrmion. Thus, the FM skyrmion will be unable to pass through the notch for all SOT strengths unless the direction of the Magnus force is reversed.

In Fig. 5, we illustrated the displacements of AFM and FM skyrmions along a racetrack notched with a notch size of R_n_ $=18\mathrm{ nm}$ and W_n_ $=50\mathrm{ nm}$ for various spin-polarized currents (SOTs). When skyrmion motions in AFM and FM racetracks are compared under the same spin-polarized current (15 $\times {10}^{10}\mathrm{A}/{\mathrm{m}}^{2}$), we find that the deflection in FM skyrmion is lower because SkHE plays here a positive role by reducing the notch edge forces, but at large currents above 20 $\times {10}^{10}\mathrm{A}/{\mathrm{m}}^{2}$, FM skyrmion collides with the edges and is destroyed.

**Figure 6:**
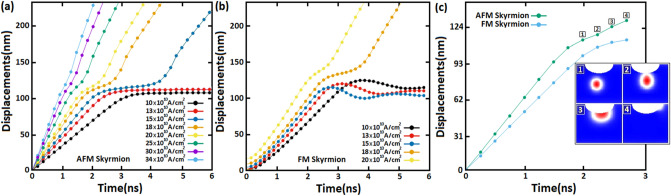
Displacements of (**a**) AFM and (**b**) FM skyrmions by various spin-polarized currents (SOTs) along the racetrack memories with notch sizes of ${\mathbf{R}}_{{\varvec{n}}}$=25 nm and (**c**) ${\mathbf{R}}_{{\varvec{n}}}$=30 nm.

On the other hand, the threshold spin-polarized current densities used to overcome the notch edge forces are related to notch size hence in Fig. 6, we reported the impact of notch radius (${\mathrm{R}}_{n})$ on displacement of AFM and FM skyrmions. Figure 6 shows the displacement of AFM skyrmion (Fig. 6a) and FM skyrmion (Fig. 6b) along racetrack memories with a notch size of ${\mathrm{R}}_{n}$=25 nm for various spin-polarized currents. The results show that for low spin-polarized currents (Fig. 6a) such as 10 $\times {10}^{10}\mathrm{A}/{\mathrm{m}}^{2}$, AFM skyrmion annihilates at the notch region instead of being blocked as for ${\mathrm{R}}_{n}$=18 nm. A similar behavior has been reported for the current density of 13 $\times {10}^{10}\mathrm{A}/{\mathrm{m}}^{2}$ which indicates that the induced SOTs are insufficient against the repulsive forces. Despite the presence of these strong forces $,$ we notice that under spin-polarized current densities greater than 13 $\times {10}^{10}\mathrm{A}/{\mathrm{m}}^{2}$, the AFM skyrmion is effectively passed through by large SOTs; nonetheless, the skyrmion motion is also accompanied by severe deflections in the notch region. For FM skyrmion (Fig. 6b), we found that at ${\mathrm{R}}_{n}$=25 nm, the SOTs induced by low spin-polarized currents of 10 $\times {10}^{10}\mathrm{A}/{\mathrm{m}}^{2}$, 13 $\times {10}^{10}\mathrm{A}/{\mathrm{m}}^{2}$, and 15 $\times {10}^{10}\mathrm{A}/{\mathrm{m}}^{2}$ are not enough to overcome the repulsive forces at the notch, because the produced SkHE does not help to sufficiently reduce the notch edge forces. However, by increasing the spin-polarized current to 18 $\times {10}^{10}\mathrm{A}/{\mathrm{m}}^{2}$ and 20 $\times {10}^{10}\mathrm{A}/{\mathrm{m}}^{2}$, we were able to exceed the notch but with deflections larger than those observed in the AFM skyrmion. We deduce that the cases where the FM skyrmion passes through are narrowed either by large currents due to the strong SkHE which drives the skyrmion towards the track edges, or by large notch sizes that generate enormous repulsive forces. In Fig. 6c, the notch radius was increased into ${\mathrm{R}}_{n}$=30 nm, then we applied a current density of 20 $\times {10}^{10}\mathrm{A}/{\mathrm{m}}^{2}$. The results reveal that there is a threshold of the notch radius where the edge forces dominate at the notch, rendering SOTs ineffective, and hence skyrmions destroy at the notch.

In Fig. 5, we observed that the SHE drives the FM skyrmion only to the bottom edge of the racetrack memory. Nevertheless, the FM skyrmion can also be stopped or destroyed at the top edge (notch) if the notch size is increased at certain large dimensions as shown in Fig. 6. We point out that the Magnus force direction can be controlled by changing the sign of the skyrmion Hall angle used or reversing the direction of the applied current density along the HM layer.

**Figure 7:**
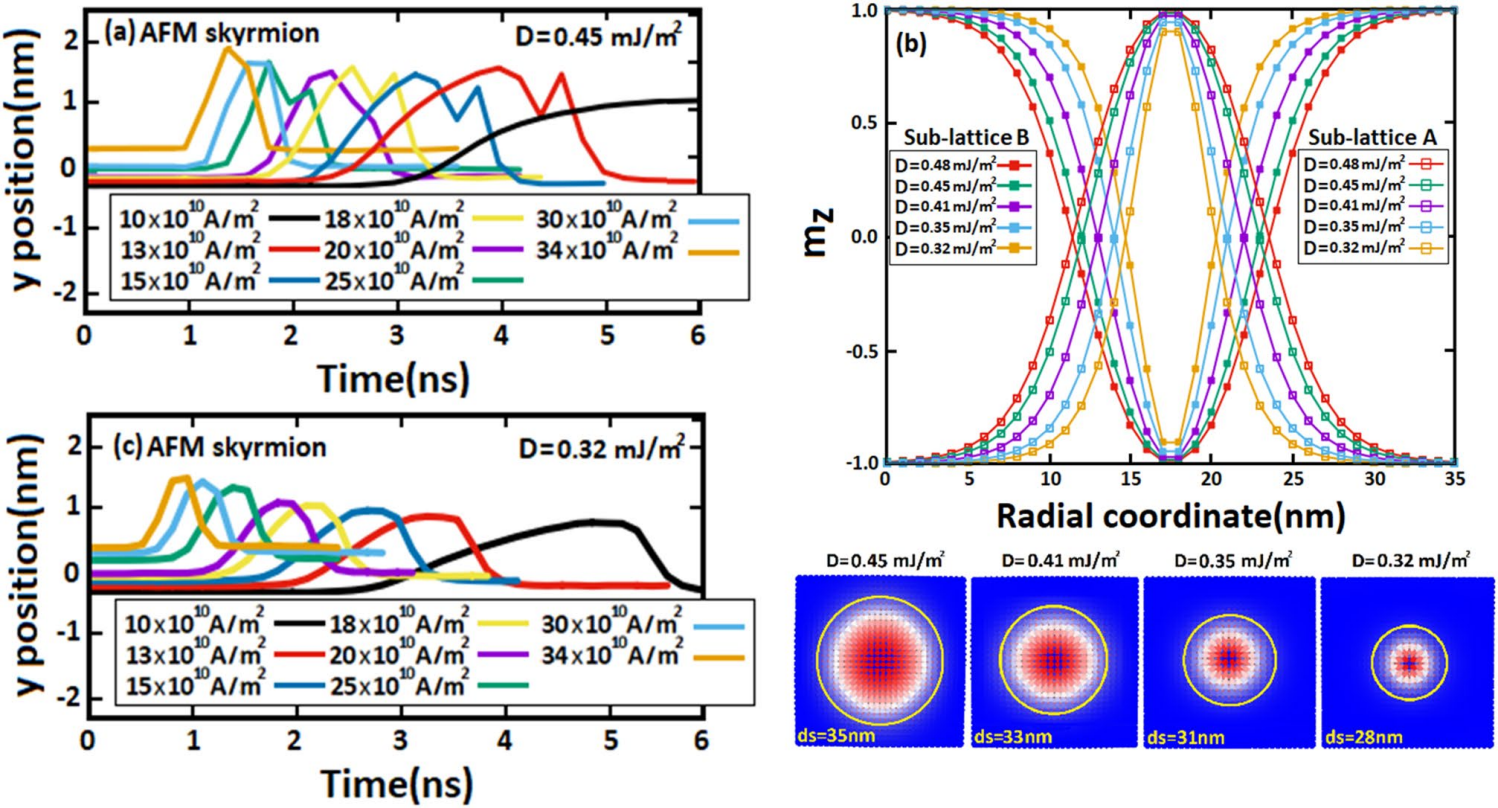
Temporal evolution of (**a,c**) AFM skyrmion position along the y-axis for different DMi strengths in a racetrack memory. (**b**) Out-of-plane magnetization along the z-axis in the sub-lattice (A) and sub-lattice (B) as a function of the radial coordinate.

**Figure 8:**
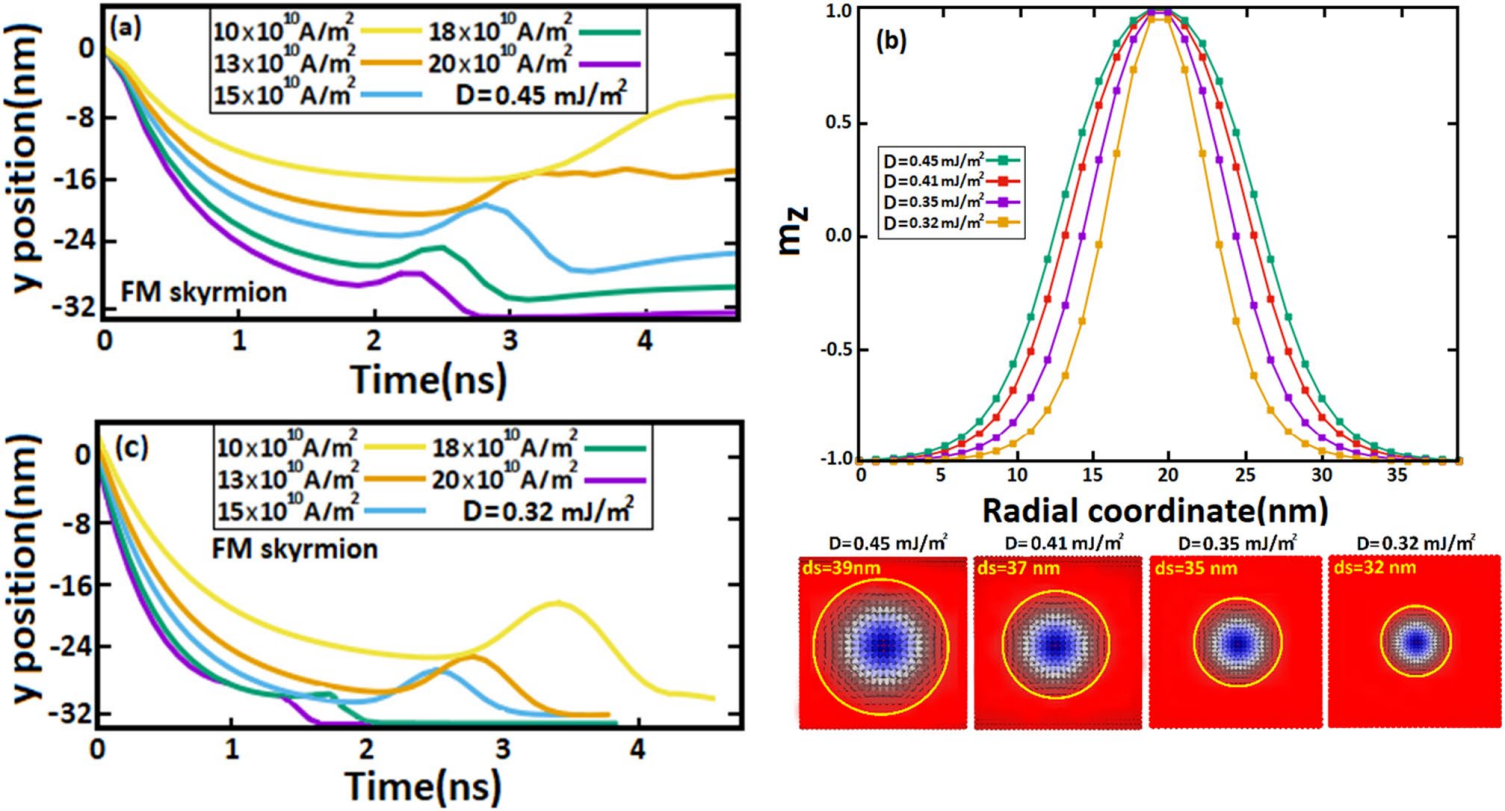
Temporal evolution of (**a,c**) skyrmion position along the y-axis for different DMi strengths in a FM racetrack notched with notch sizes of ${\mathbf{R}}_{{\varvec{n}}}$=18 nm and ${\mathbf{W}}_{\mathbf{n}}$=50 nm. (**b**) Out-of-plane magnetization in the z-axis along the FM skyrmion.

To elucidate the major impact of SkHE on skyrmion displacements, we studied in Figs. 7 and 8, the evolution of skyrmion positions along the y-axis in AFM and FM racetrack memories for different DMi strengths and varied SOTs. Figure 7a shows that AFM skyrmion positions along the y-axis do not change because SkHE does not affect skyrmion motion ($${\theta }_{SkHA}$$~$$0)$$ since $${Q}_{AFM}=0$$, therefore the AFM skyrmion continues to move steadily along the x-axis under SOTs. The resulting peaks are due to the interaction between the skyrmion and notch, where next to the notch, the AFM skyrmion is repulsed towards the notch by edge forces, thereby the skyrmion position increases somewhat along the y-axis. If the SOTs applied are large enough, the skyrmion can bypass the notch, therefore this interaction can be decreased again with the skyrmion motion. While for low spin-polarized current of 10 $$\times {10}^{10}\mathrm{A}/{\mathrm{m}}^{2}$$, the skyrmion stops on the left side of the notch and as we see, no peak is shown. In Fig. 7c, we decreased the DMi strength to 0.32 mJ/m^2^ subsequently we repeated the same process. The results show that the peaks were decreased compared to those at 0.45 mJ/m^2^ which indicates that the repulsive forces are decreased as well. This reduction is essentially due to the skyrmion size which decreases with the DMi strength (D), allowing SOTs to efficiently manipulate the skyrmion motion in the notch. As a result, succeeded to overcome the notch edge forces with a relatively low spin-polarized current of 10 $$\times {10}^{10}\mathrm{A}/{\mathrm{m}}^{2}$$, and hence a wide peak was shown. The size variation of AFM skyrmion as a function of DMi strengths is provided in Fig. 7b by plotting the out-of-plane magnetization along the z-axis either in the sub-lattice B which is characterized by a negative skyrmion number ($${\mathrm{Q}}_{\mathrm{B}}=-1$$) or in the sub-lattice A with a positive skyrmion number ($${\mathrm{Q}}_{\mathrm{A}}=+1$$). Indeed, analyzing the skyrmion behavior in this range of DMi strength was not arbitrary, as we tested various DMi strengths. As a result, we found that, according to the notch size used ($${\mathrm{R}}_{n}$$=18 nm and $${\mathrm{W}}_{n}$$=50 nm), the nucleation of AFM skyrmions by using DMi strengths of 0.47$$-$$0.32 mJ/m^2^ displays adequate skyrmion sizes allowing us to investigate the skyrmion flexibility in the notch. For small DMi strengths, the results were very predictable since skyrmions stabilize with small sizes, and practically no interaction showed at the notch. For strong DMi, large sizes can be shown. Therefore, all skyrmions annihilate at the notch.

For FM skyrmion, Fig. 8a shows that the skyrmion position along the y-axis is significantly displaced as compared to the AFM skyrmion due to the SkHE which strongly drives the skyrmion motion under large SOTs to deviates from its longitudinal motion. However, the SkHE may be ignored for low spin-polarized currents of 10 $\times {10}^{10}\mathrm{A}/{\mathrm{m}}^{2}$ and 13 $\times {10}^{10}\mathrm{A}/{\mathrm{m}}^{2}$, therefore it does not assist to minimize the repulsive forces. Thus, FM skyrmion is unable to pass through. However, for sufficiently large SOTs induced by 15 $\times {10}^{10}\mathrm{A}/{\mathrm{m}}^{2}$, 18 $\times {10}^{10}\mathrm{A}/{\mathrm{m}}^{2}$ and 20 $\times {10}^{10}\mathrm{A}/{\mathrm{m}}^{2}$, SkHE is significantly developed, therefore the transverse component of velocity pulls the skyrmion away from the notch along the y-axis which somehow reduces the interaction between the skyrmion and notch and allows the skyrmion to exceed the notch. However, for large SOTs, SkHE leads the FM skyrmion to annihilate at the track boundary because of the strong transverse velocity component. Therefore, we infer that the skyrmion can thus exceed the notch in the FM racetrack memory only in a narrow range of spin-polarized currents where SkHE begins to develop. Besides, the smaller the skyrmion size, the larger the SkHE, therefore by decreasing the DMi strengths (Fig. 8b), the FM skyrmion size also decreases which increases the SkHE during the skyrmion motion. Consequently, we observe in Fig. 8c that at a small DMi strength of 0.32 mJ/m^2^, the skyrmion size decreases into 32 nm thus the transverse velocity component drives the FM skyrmion strongly along the y-axis to be destroyed at the side edge. As a result, the previously indicated range of spin-polarized currents, allowing FM skyrmion to minimize repulsive forces in the notch region becomes even smaller for small skyrmion sizes due to the SkHE. In short, we deduce that for large sizes of FM skyrmion, the repulsive forces are significant in the notch, hindering the skyrmion motion. Moreover, SkHE develops crucially for small skyrmion sizes, leading FM skyrmions to be annihilated at the side edge. On the contrary, for AFM skyrmion (see Fig. 7), the SkHE does not exist during the skyrmion motion, furthermore at small sizes, SOTs drive skyrmions to overcome the boundary forces in the notch for both low and large spin-polarized pulses which cannot be achieved for FM skyrmion due to the SkHE and repulsive forces.

**Figure 9:**
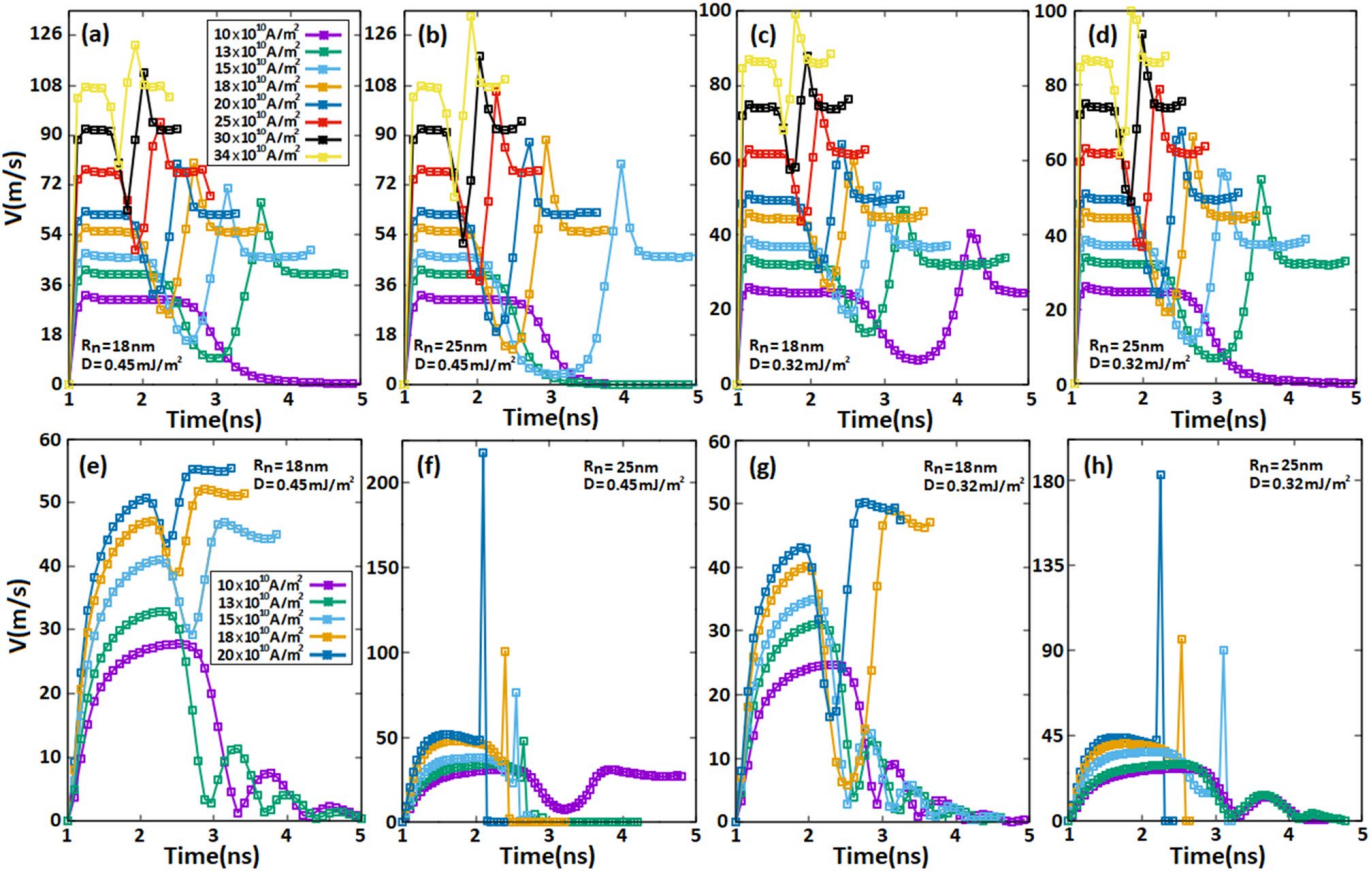
Temporal evolution of (**a–d**) the AFM skyrmion velocity and (**e–h**) the FM skyrmion velocity for various SOTs along the racetrack memory with different skyrmion and notch sizes.

We present in Fig. 9 a comprehensive comparison between the velocity of AFM and FM skyrmions for various SOTs along racetrack memories with different skyrmion and notch sizes. As shown in Fig. 9a, we adjusted the notch size at ${\mathrm{R}}_{n}=$ 18 nm and the skyrmion size to ${\mathrm{d}}_{\mathrm{sk}}=$ 35 nm, and then various spin-polarized currents were applied. For a spin-polarized current of 10 $\times {10}^{10}\mathrm{A}/{\mathrm{m}}^{2}$, the AFM skyrmion velocity increases at the beginning to its maximum value at 26.2 m/s thereafter, skyrmion moves with a steady velocity along the racetrack. However, as the skyrmion approaches to the notch, the velocity decreases until it vanishes completely next to the notch. The maximum value of the velocity increases with spin-polarized currents used, thus we notice that the AFM skyrmion exceeds the notch but with drastic velocity decelerations in the notch region. However, these velocity decelerations can be effectively reduced for large spin-polarized currents. In Fig. 9b, we increased the notch size to 25 nm while keeping the skyrmion size constant at 35 nm. Due to the large notch, the repulsive forces are increased as well, thus we observe that the velocities associated with spin-polarized currents of 10 $\times {10}^{10}\mathrm{A}/{\mathrm{m}}^{2}$ and 12 $\times {10}^{10}\mathrm{A}/{\mathrm{m}}^{2}$ collapses closer to the notch. Furthermore, the velocity dramatically reduced to approximately zero for 15 $\times {10}^{10}\mathrm{A}/{\mathrm{m}}^{2}$ before increasing again to 13.24 m/s. Whereas for large spin-polarized currents, similar behaviors were found compared to the Fig. 9a, except that the velocity decelerations in the notch are stronger than before due to the large notch size. Figure 9c shows the impact of skyrmion size on skyrmion velocity in an AFM racetrack memory by decreasing the skyrmion size into 28 nm and fixing the notch size at 18 nm. The results proved that for small skyrmion sizes, the skyrmion velocities decrease and the repulsive forces reduce, and hence the ability to exceed the notch increases. Thus, even under a spin-polarized current of 10 $\times {10}^{10}\mathrm{A}/{\mathrm{m}}^{2}$, SOTs can lead the skyrmion to pass through with velocity decelerations lower than those at ${\mathrm{d}}_{\mathrm{sk}}= 35\mathrm{ nm}$. Figure 9d shows similar behaviors, where at a large notch size of ${\mathrm{R}}_{n}=$ 25 nm, we are able to exceed the notch under a low spin-polarized current of 12 $\times {10}^{10}\mathrm{A}/{\mathrm{m}}^{2}$ for a skyrmion size of 28 nm. As for large spin-polarized currents, AFM skyrmion successfully bypassed the notch with minimal velocity decelerations in the notch. On the contrary, for FM skyrmion (Fig. 9e–h), the velocity exhibits different behaviors compared to the AFM skyrmion; SkHE grows as skyrmion size decreases, hence SHE forcefully drives skyrmion to the side edge. As a result, we observe that the velocity abruptly drops to zero as shown in Fig. 9g,h. However, for large sizes of FM skyrmion (Fig. 9e,f), the repulsive forces cancel all velocities under low spin-polarized currents either by blocking skyrmion if the notch size is small enough (Fig. 9e) or destroying skyrmion for large notch sizes (Fig. 9f). The results lucidly showed that for the AFM skyrmion in Fig. 9a–d, most of the SOTs drive skyrmion to exceed the notch area with different deceleration velocities in the notch according to the SOT value and notch size. In contrast, we saw in Fig. 9e–h that due to the SkHE and repulsive forces, FM skyrmion can pass through only in a few situations illustrated in Fig. 9e,g where the spin-polarized current and the skyrmion size are small enough which is not recommended in the new generation of racetrack memories.

**Figure. 10:**
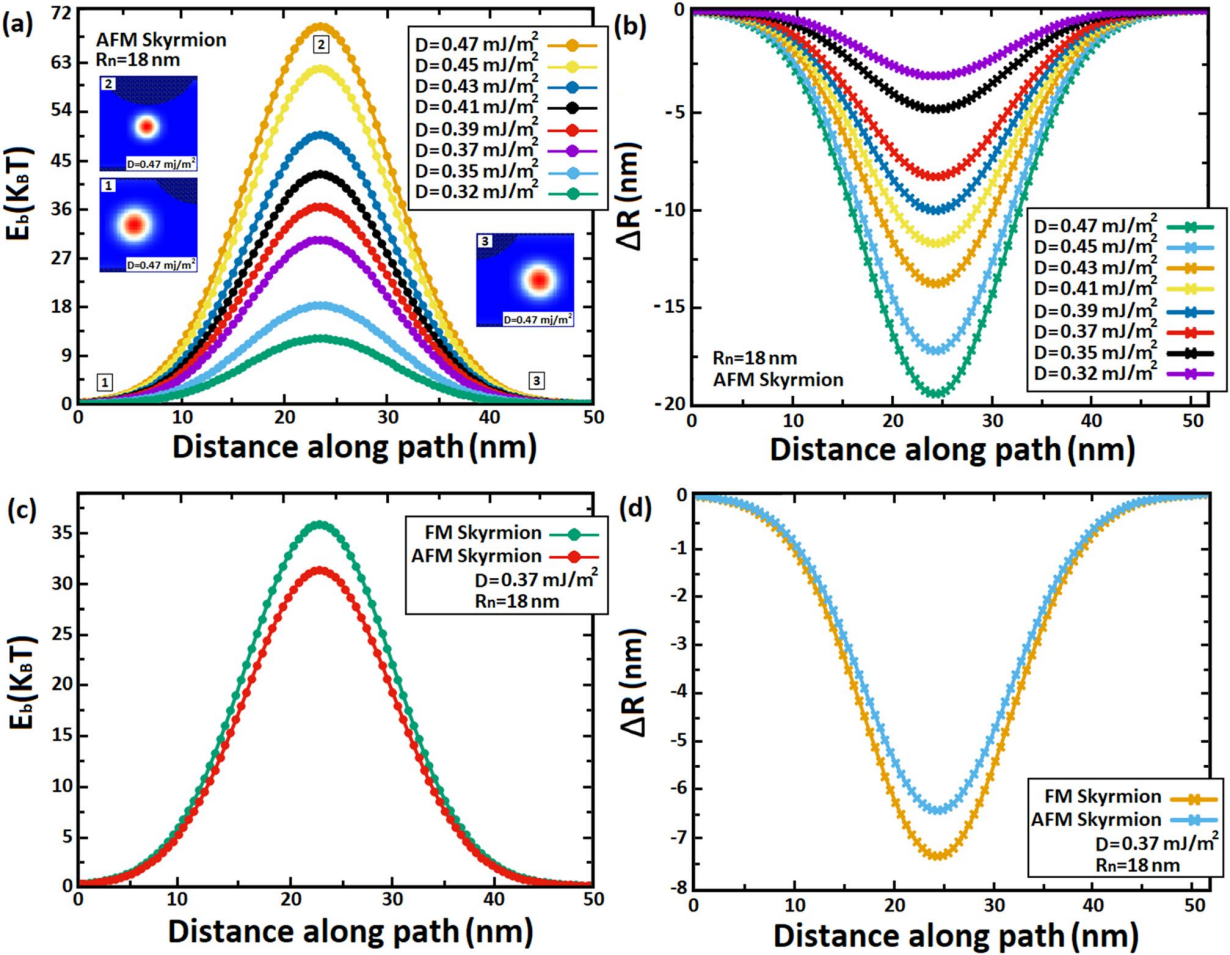
(**a,c**) Variations of the energy barrier (E_b_) in the notch region for AFM and FM skyrmions with various skyrmion sizes R_sk_ in a racetrack memory notched with a fixed notch size of ${\mathbf{R}}_{{\varvec{n}}}$ $=$ 18 nm and ${\mathbf{W}}_{{\varvec{n}}}=$ 50 nm. (**b,d**) Shrinkage of skyrmions (∆R_sk_) along the notch width (${\mathbf{W}}_{{\varvec{n}}}$).

In Fig. 10, we carried out the variation of energy barrier (∆E_b_) when an AFM and FM skyrmions pass through a notch with a width of 50 nm (${\mathrm{W}}_{\mathrm{n}}$) using the minimum energy path (MEP) calculations. Every stable magnetic state corresponds to a local minimum on the energy surface that characterizes the magnetic structure^46^. The minimum-energy path (MEP) is defined as a path that connects these local minima to plot the lowest-energy path on a potential energy surface. Local minima can be calculated by using several methods such as the Conjugate Gradient^47^, Nudged Elastic Band^46^, String Method^48^ and Synchronous Transit^49^ to find the minimum energy path between metastable magnetic states and therefore calculate the energy barrier. In this work, we adopted the String method, which works by guessing the MEP which connects the metastable skyrmions (so-called images) when the skyrmion crosses the notch. Additionally, the images are moved in short distances along the MEP to ensure that they are appropriately spaced according to the force acting on skyrmions^48^.

We used MEP calculations to obtain the total energy variation in the notch area. When the skyrmion penetrates the notch, the local energy of the metastable skyrmion increases gradually by exchange energy, DMi energy, and anisotropy energy which varied according to the variation of the skyrmion size (∆R_sk_) in the notch as shown in Fig. 10: the more the skyrmion gets into the notch, the narrower the notch becomes, and hence repulsive forces squeeze more and more the skyrmion in the notch, as a result skyrmion shrinks progressively which increases gradually the local energy until it reaches its maximum value at the center of the notch. This skyrmion behavior imposes an energy barrier at the notch area since the local maximum energy depends on the skyrmion size, thus skyrmions can pass through the notch only for certain values of energy according to the skyrmion size (DMi strength) and notch size. In Fig. 10a, we calculated the energy barrier associated with the AFM skyrmion in the notch using the MEP calculations for various skyrmion sizes. Moreover, we plotted in Fig. 10b, the skyrmion size variations ∆R_sk_ according to the acquired energy barriers during the skyrmion penetration along the path. Our findings show that the larger the skyrmion size, the harder it is for the skyrmion to shrink further in the notch, resulting in a significant energy barrier E_b_. Thus, for small skyrmion sizes, skyrmion generates low energy barriers which explain why low spin-polarized currents may lead the AFM skyrmion to penetrate the notch area. While for large skyrmions, the energy barrier is high because skyrmions need to shrink more than small skyrmions to pass through the notch which produces high energy. If we increase the notch size or use a very large skyrmion size, significant values can be exhibited, and hence the unstable skyrmions can be destroyed at the notch. In Fig. 10c,d, we compared the variations in energy barrier and skyrmion size between the AFM and FM skyrmions for the same notch size (${\mathrm{R}}_{n}$ = 18 nm and ${\mathrm{W}}_{n}$=50 nm). The results indicate that the energy barrier of AFM skyrmion is smaller than that of FM skyrmion (Fig. 10c) which elucidates the need for additional energy (SOTs) in the FM skyrmion to exceed the notch. These results are clearly explained by Fig. 10d, wherein we found out that the skyrmion shrinkage in the FM skyrmion is large compared to the AFM skyrmion, thereby the AFM skyrmion can pass over the notch with SOTs smaller than those used in the FM skyrmion. We point out that for FM skyrmions, large current densities induce strong SkHEs, thus energy barriers cannot be plotted.

**Figure 11:**
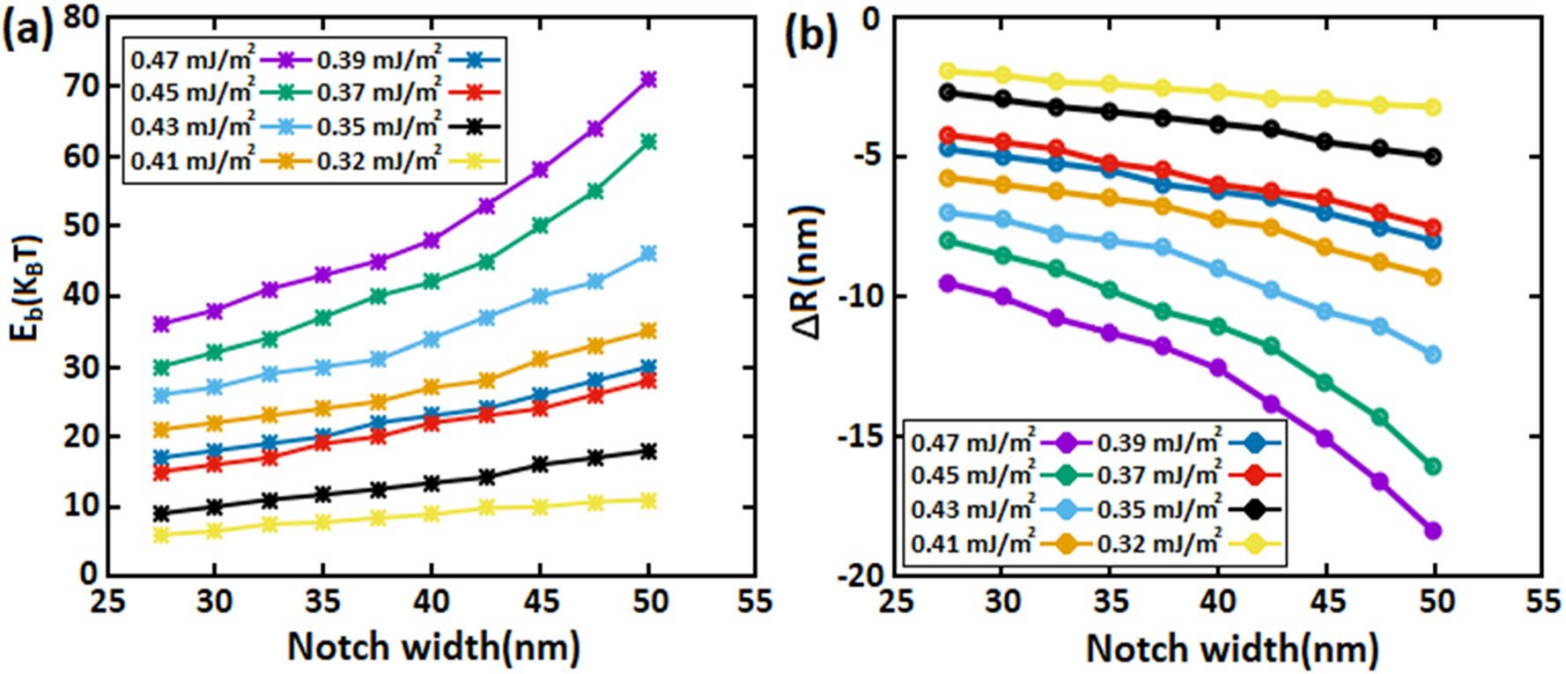
Energy barrier and skyrmion radius as a function of notch width (W_n_) for various skyrmion sizes.

In Fig. 11a, we calculated the maximum values of the energy barrier E_b_ and skyrmion size (R_sk_) in each notch width (W_n_) for various DMi strengths. The results show that the wider the notch width, the higher the energy barrier as well as the energy barrier increases as the skyrmion size reduces, consistent with the Fig. 11b. For wide W_n_, the skyrmion size shrinks further, and ∆R_sk_ gets larger therefore E_b_ increases proportionally. Furthermore, when notch width surpasses 50 nm, the skyrmion annihilates at the notch because the notch area becomes insufficient to allow the skyrmion to pass through.

## Conclusion

To summarize, we numerically investigated the nucleation of an isolated anti-ferromagnetic (AFM) by locally injecting a pure spin current perpendicularly to the 2D AFM layer via spin-transfer torques (STTs). Besides, we revealed the key advantage of hosting skyrmion in an AFM racetrack memory by displaying a comprehensive comparison between the motion of AFM and FM skyrmions with different radii using spin–orbit torques (SOTs) along a racetrack memory notched with various notch sizes. As a result, we observed that for large FM skyrmion sizes, repulsive forces inhibit the skyrmion motion, while for small sizes, SkHE grows significantly with SOTs which drives the skyrmion to be destroyed at the side edge. In contrast, for AFM skyrmion, SkHE does not exist during the skyrmion motion, therefore at small skyrmion sizes, we succeeded to overcome the repulsive forces at low and high SOTs that cannot be achieved for FM skyrmion. We also investigated the impact of notch size on skyrmion motion by increasing the notch radius (${\mathrm{R}}_{\mathrm{n}}$) to 25 nm. Thus, we found that the AFM skyrmion is still able to pass through the notch under large SOTs. Whereas for FM skyrmion, the skyrmion is either blocked under low SOTs or utterly destroyed in the track boundary under large SOTs due to strong Magnus forces. Furthermore, we calculated the variation of energy barrier and skyrmion size in the notch region using MEP. Specifically, we established that the larger the skyrmion size, the more difficult it is to shrink the skyrmion through, which generates a high energy barrier (E_b_) in the notch. For wide notches (W_n_), the skyrmion size shrinks further and therefore E_b_ increases proportionally. In perspective, we plan to achieve an ultrafast nucleation time of less than 5.5 ps as well as reduce the velocity decelerations in the notch, which would be crucial for the development of new AFM skyrmion-based track memories.

## Supplementary Information

Supplementary Information.

## Data Availability

All data generated or analysed during this study are included in this published article and its supplementary information file.
