# Supplementary material for: Nucleation and manipulation of single skyrmions using spin-polarized currents in antiferromagnetic skyrmion-based racetrack memories
Source: Sci Rep. 2022 Sep 8;12:15225. doi: 10.1038/s41598-022-19587-6 (PMC9458664; doi:10.1038/s41598-022-19587-6)
Supplement: Supplementary file 1 — Supplementary Information. [file 41598_2022_19587_MOESM1_ESM.docx]

**Supplementary file:**

Article: **Nucleation and manipulation of single skyrmions using spin-polarized currents in antiferromagnetic skyrmion-based racetrack memories**

Authors: Hamza BELRHAZI & Mohamed EL HAFIDI

1. **Supplementary Movies**

**Movie 1:** **AFM monolayer**

**Link:** <https://drive.google.com/file/d/1ia2Opi-wv-lYy2mXKgei_ZWvJxPjKmrY/view?usp=sharing>

**Movie 2:** **Nucleation of AFM skyrmion via STT**

**Link:** <https://drive.google.com/file/d/1pxlGKK0fKr_MBm7xlSeAhQmzJWPz066a/view?usp=sharing>

**Movie 3:** **AFM skyrmion in the track memory**

**Link:** <https://drive.google.com/file/d/1hNSb1dO_eEl27zodrQJbeh54aQrwfA6O/view?usp=sharing>

**Movie 4:** **Displacement of AFM skyrmion along a racetrack memory via SOT**

**Link:** <https://drive.google.com/file/d/1v_sGG7G-S1lT18ln3Vrpg67jB4HGB7oo/view?usp=sharing>

**Movie 5:** **Periodic boundary effect**

**Link:** <https://drive.google.com/file/d/1YDe7i7BE1Nat1oUmDHcf80QqQTUgR-3K/view?usp=sharing>

1. **Movie Supplementary Tables**

**Movie 2:** **Nucleation of AFM skyrmion via STT**

**Link:** <https://drive.google.com/file/d/1kG0GLaP1k6xH4O54cWfi9_1ETx-Hn-fW/view?usp=sharing>

**Movie 4:** **Displacement of AFM skyrmion along a racetrack memory via SOT**

**Link:** <https://drive.google.com/file/d/1ccDQ_Z1MIOmg5bamNe4L6vLcITnZj7Zm/view?usp=sharing>

**Movie 5:** **Periodic boundary effect**

**Link:** <https://drive.google.com/file/d/1U5m8NOGiDdl-kY1zvfRTheymrVbIg6o2/view?usp=sharing>

1. **Supplementary Tables**

**Figure.3: (a) Snapshots of the nucleation steps of isolated AFM skyrmion using a vertically injected spin-polarized current (STT) perpendicular-to-plane (CPP) of the 2D AFM layer in a circular region (yellow circular). (b) The time evolution of topological charge QA and QB during the nucleation. (c and d) Snapshots of isolated AFM and FM skyrmions after the nucleation time**

**Link:** <https://drive.google.com/file/d/1a3Bt034XUZxWJKpG9QFJhDdsIUs6kQV_/view?usp=sharing>

**Figure.4: Displacements of (a) AFM and (b) FM skyrmions by various spin-polarized currents (SOTs) along racetrack memories with a notch size of R_n_= 18 nm and W_n_ = 50 nm.**

**Link:** <https://drive.google.com/file/d/13lMTgvMWoabsD-FxoGOAz8hloUyBUQH5/view?usp=sharing>

**Figure.6:** **Displacements of (a) AFM and (b) FM skyrmions by various spin-polarized currents (SOTs) along the racetrack memories with notch sizes of R_n_ =25 nm and (c) R_n_ =30 nm.**

**Link:** <https://drive.google.com/file/d/17GNOYLbbqc3y_vwQ4I943hCnGyydRm5u/view?usp=sharing>

**Figure.7:** **Temporal evolution of (a and c) AFM skyrmion position along the y-axis for different DMi strengths in a racetrack memory. (b) Out-of-plane magnetization along the z-axis in the sub-lattice (A) and sub-lattice (B) as a function of the radial coordinate.**

**Link:** <https://drive.google.com/file/d/1V1AYO-mn2n6jeAvv5qDCZUmn20cCEk9e/view?usp=sharing>

**Figure.8:** **Temporal evolution of (a and c) skyrmion position along the y-axis for different DMi strengths in a FM racetrack notched with notch sizes of R_n_ =18 nm and W_n_ =50 nm. (b) Out-of-plane magnetization in the z-axis along the FM skyrmion.**

**Link:** <https://drive.google.com/file/d/177g3tL5va_nmzXq1zbccrDp7b56gdoR_/view?usp=sharing>

**Figure.9: Temporal evolution of (a, c, d, and d) the AFM skyrmion velocity and (e, f, g and h) the FM skyrmion velocity for various SOTs along the racetrack memory with different skyrmion and notch sizes.**

**Link:** <https://drive.google.com/file/d/12fmNULtd9Pfy8QyfU-nfkrpoXtw3BIgm/view?usp=sharing>

**Figure.10:** **(a and c) Variations of the energy barrier (Eb) in the notch region for AFM and FM skyrmions with various skyrmion sizes Rsk in a racetrack memory notched with a fixed notch size of R_n_ = 18 nm and W_n_= 50 nm. (b and d) Shrinkage of skyrmions (∆Rsk) along the notch width (W_n_).**

**Link:** <https://drive.google.com/file/d/1XFQ5UOeSEV77eGIHWmjsB_f5rGijc2eV/view?usp=sharing>

**Figure.11:** **Energy barrier and skyrmion radius as a function of notch width (Wn) for various skyrmion sizes**

**Link:** <https://drive.google.com/file/d/1LJZYM5jVoy3UMlE4jwO8KymjuNdtOTtn/view?usp=sharing>

1. **All datasets**

**Link:** <https://drive.google.com/file/d/1obz_POA_ocv2R8wsAAJSwGKAcuy5DeKE/view?usp=sharing>
